# Norepinephrine-Activated p38 MAPK Pathway Mediates Stress-Induced Cytotoxic Edema of Basolateral Amygdala Astrocytes

**DOI:** 10.3390/brainsci14020161

**Published:** 2024-02-04

**Authors:** Zhaoling Sun, Xiaojing Zhang, Yiming Dong, Yichang Liu, Chuan Wang, Yingmin Li, Chunling Ma, Guangming Xu, Songjun Wang, Chenteng Yang, Guozhong Zhang, Bin Cong

**Affiliations:** 1 Hebei Key Laboratory of Forensic Medicine, Collaborative Innovation Center of Forensic Medical Molecular Identification, College of Forensic Medicine, Hebei Medical University, Shijiazhuang 050017, China; szl503743400@163.com (Z.S.); 17800745@hebmu.edu.cn (X.Z.); dym6127569@163.com (Y.D.); 95417026@163.com (C.W.); 16000557@hebmu.edu.cn (Y.L.); chunlingma@hebmu.edu.cn (C.M.); fyguangming@163.com (G.X.); 17800584@hebmu.edu.cn (S.W.); chn_yct4750@hebmu.edu.cn (C.Y.); 2 Department of Forensic Medicine, College of Medicine, Nantong University, Nantong 226000, China; lyc2008@ntu.edu.cn; 3 Hebei Province Laboratory of Experimental Animal, Shijiazhuang 050017, China; 4 Hainan Tropical Forensic Medicine Academician Workstation, Haikou 571199, China

**Keywords:** stress, basolateral amygdala, astrocyte, cytotoxic edema, AQP4, p38 MAPK pathway

## Abstract

The amygdala is a core region in the limbic system that is highly sensitive to stress. Astrocytes are key players in stress disorders such as anxiety and depression. However, the effects of stress on the morphology and function of amygdala astrocytes and its potential mechanisms remain largely unknown. Hence, we performed in vivo and in vitro experiments using a restraint stress (RS) rat model and stress-induced astrocyte culture, respectively. Our data show that norepinephrine (NE) content increased, cytotoxic edema occurred, and aquaporin-4 (AQP4) expression was up-regulated in the basolateral amygdala (BLA) obtained from RS rats. Additionally, the p38 mitogen-activated protein kinase (MAPK) pathway was also observed to be significantly activated in the BLA of rats subjected to RS. The administration of NE to in vitro astrocytes increased the AQP4 level and induced cell edema. Furthermore, p38 MAPK signaling was activated. The NE inhibitor alpha-methyl-p-tyrosine (AMPT) alleviated cytotoxic edema in astrocytes, inhibited AQP4 expression, and inactivated the p38 MAPK pathway in RS rats. Meanwhile, in the in vitro experiment, the p38 MAPK signaling inhibitor SB203580 reversed NE-induced cytotoxic edema and down-regulated the expression of AQP4 in astrocytes. Briefly, NE-induced activation of the p38 MAPK pathway mediated cytotoxic edema in BLA astrocytes from RS rats. Thus, our data provide novel evidence that NE-induced p38 MAPK pathway activation may be one of the mechanisms leading to cytotoxic edema in BLA under stress conditions, which also could enable the development of an effective therapeutic strategy against cytotoxic edema in BLA under stress and provide new ideas for the treatment of neuropsychiatric diseases.

## 1. Introduction

Due to rapid developments in the modern world, long-term, persistent mental and environmental stresses affect the physical and mental health of millions of people globally. Appropriate stress can increase the body’s ability to adapt and cope with environmental changes [1]; however, excessive or persistent stress can cause acute or chronic organ dysfunction and metabolic disorders [2]. Many studies [3,4,5] have found that long-term persistent stress can lead to many neuropsychiatric disorders, such as anxiety, depression, epilepsy, schizophrenia, etc. It is extremely important to study the pathological mechanisms by which stress factors induce neuropsychiatric disorders in order to achieve precise diagnosis and appropriate treatments for stress-related neuropsychiatric disorders.

As a part of the limbic system, the amygdala is highly sensitive to stress and plays a critical role in regulating stress-induced central nervous system disorders [6]. Dysregulation of the amygdala participates in the pathogenesis of psychiatric disorders such as depression and anxiety [7]. Previous studies on amygdala damage under stress have mainly focused on morphological and functional changes to neurons [8,9], and there have been few studies on the morphological and functional changes of astrocytes under stress. Astrocytes are the most common neuroglial cells in the central nervous system, whose functions include promoting formation of the blood–brain barrier, supporting and protecting neurons, regulating synapse formation and function, and maintaining cerebral aqueous homeostasis and regulation of central nervous system (CNS) inflammation [10]. Pathological astrocyte swelling can have severe adverse effects on neuronal function [11]. An increasing body of evidence [12,13,14] has shown that astrocytes participate in the formation and pathological remodeling of mood disorders and stress-related diseases. Therefore, it is necessary to target astrocytes in order to further understand the biological underpinnings of these diseases and potentially reveal novel therapeutic avenues. However, whether or not stress induces cytotoxic edema in astrocytes and its potential mechanisms remain largely unknown.

As an important neurotransmitter in the central nervous system, norepinephrine (NE) is involved in a variety of stress-related diseases [15,16,17]. Evidence has indicated that restraint stress can increase NE levels in the amygdala [18]. It is worth mentioning that NE plays an important role in the clearance of neurotransmitters, nutritional support of neurons, and immunomodulatory functions of astrocytes [19]. No papers to date have reported whether astrocyte cytotoxic edema under excessive stress is related to NE.

Mitogen-activated protein kinases (MAPKs) are serine/threonine protein kinases that regulate the cell cycle, cell proliferation, inflammation, and many physiological and pathological processes through intracellular signaling [20,21]. Studies [22,23] have shown that the p38 MAPK signaling pathway plays an important role in mediating the biological responses to NE. Other studies have demonstrated the regulation of aquaporins (AQPs) by p38 MAPK pathway activation in cerebral edema. Based on the above research background, we considered whether NE activation of the p38 MAPK pathway mediates the pathological process of amygdala astrocyte edema under stress. 

In this study, to investigate the role of NE in stress-induced cytotoxic edema in basolateral amygdala (BLA) astrocytes, we first established a well-recognized, depression-like model through the exposure of male rats to chronic restraint stress (CRS). CRS can be used to establish mature animal models of anxiety and depression which simulate the daily annoyance and stress levels of humans, and this is considered an ideal animal model for this purpose [24]. We found that NE activated the p38 MAPK pathway and mediated cytotoxic edema in BLA astrocytes in RS rats. Second, the selective p38 MAPK antagonist SB203580 reversed NE-induced cytotoxic edema and down-regulated the expression of aquaporin-4 (AQP4) in primary culture astrocytes. Our data provide novel evidence that inhibiting the NE/p38 MAPK pathway represents an attractive approach for the rescue of stress-induced cytotoxic edema in BLA astrocytes. The results of the present study elucidate the relationship between the morphological basis and potential pathological mechanisms of stress-induced cytotoxic edema in BLA astrocytes, and provide new ideas for the treatment of neuropsychiatric diseases.

## 2. Materials and Methods

### 2.1. Experimental Animals

Male Sprague–Dawley rats weighing 220 ± 10 g were purchased from Beijing Vital River Laboratory Animal Technology Co., Ltd. (Beijing, China). All rats were provided with food and water ad libitum, and were kept in a climate-controlled environment at a consistent temperature (22 ± 2 °C), humidity (60–65%), and 12 h light/dark cycle. Rats were randomized into a control group, a stress 1-day group, a stress 7-day group, a stress 14-day group, and a stress 21-day group. In addition, in order to study the effects of NE on stress-induced cytotoxic edema in BLA astrocytes, we also included a group exposed to stress that was treated with the NE synthesis rate-limiting enzyme tyrosine hydroxylase inhibitor alpha-methyl-p-tyrosine (AMPT, 120693; Sigma-Aldrich, St. Louis, MO, USA) for 7 days (AMPT + RS) and a group that was only treated with AMPT for 7 days (AMPT; *n* = 5 rats per group). All procedures followed the National Institutes of Health guidelines and were approved by the Institutional Review Board for Animal Experiments at Hebei Medical University.

### 2.2. Animal Treatments and Experimental Procedure

The model of restraint stress was established as previously described [25]. In brief, rats were placed in an adjustable acrylic cylinder for 6 h every day (from 09:00 to 15:00). Restraint stress lasted for 1, 7, 14, or 21 days. With the exception of restraint stress, the living conditions of the control group were identical to those of the stress group (*n* = 5–10 per group). For the AMPT + RS group, rats were injected intraperitoneally (i.p.) with AMPT (100 mg/kg, i.p.) 1 h before stress treatment. For the AMPT group, rats were only injected with AMPT (100 mg/kg, i.p.). For the control group, rats were only injected with normal saline (NS, i.p.). For the stress group, rats were injected with NS (i.p.) 1 h before stress treatment. The protocols for the control, stress, AMPT, and AMPT + RS groups were performed for 7 days (*n* = 5–10 per group). The drug concentration was selected according to the change in blood pressure. AMPT (100 mg/kg) was injected intraperitoneally 1 h before restraint stress, which not only reduced the level of NE in the blood, but also had little impact on the blood pressure of the rats.

### 2.3. Behavioral Experiments

A video recorder was mounted on top of an elevated plus maze (EPM). The main cross structure was black organic glass located 50 cm above the ground. An open arm (50 cm × 10 cm) and a closed arm (50 cm × 10 cm × 40 cm) were placed opposite to each other, with a 10 cm × 10 cm platform in the middle. The EPM video analysis system developed by Shanghai Jiliang Software Science and Technology Co., Ltd. (Shanghai, China) was employed to analyze the percentage (%) of entries into the open arm [number of entries in open arm/(number of entries in open arm + number of entries in closed arm)] × 100% and the percentage of time spent in the open arm (%) [time spent in the open arm/(time spent in the open arm + time spent in the closed arm)] × 100% in order to observe anxiety-like behavior in rats [26].

### 2.4. Determination of Serum and BLA Levels of NE and Epinephrine (E)

Enzyme-linked immunosorbent assay (ELISA) was used to measure serum and tissue NE concentrations. Collected rat blood was left to stand at 4 °C for 1 h, then centrifuged at 825× *g* and 4 °C for 10 min. The supernatant was collected. Rat BLA was harvested and mechanically homogenized in physiological saline. The homogenate was centrifuged at 13,201× *g* and 4 °C for 10 min, and the supernatant was collected. The serum and BLA levels of NE were determined using a 3-CAT ELISA kit (Labor Diagnostika Nord, Nordhorn, Germany) in accordance with the manufacturer’s protocol.

### 2.5. Tissue Preparation

**Figure 1:**
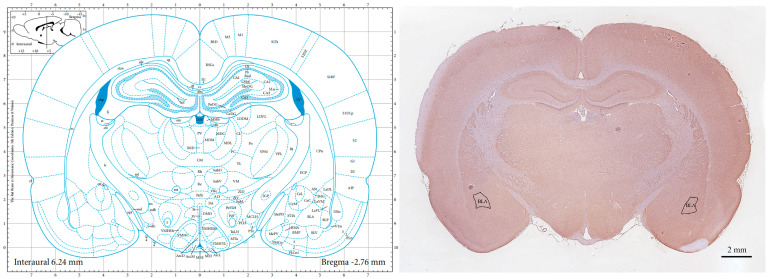
The section with the largest BLA area. BLA: basolateral amygdala.

All rats were anesthetized through intraperitoneal injection of 2% sodium pentobarbital (50 mg/kg) after the last RS was completed. Brain tissues for staining were collected and immediately fixed in 10% formalin. After ethanol gradient dehydration and paraffin embedding, the brain tissue containing the BLA (sagittal plane, −1.34 mm to −3.48 mm from the anterior fontanelle) was cut out based on the rat brain positioning map. The tissue was cut into 5 μm brain sections (Figure 1) for HE staining and immunofluorescence staining. Brain tissues were immediately collected from other rats, and the bilateral BLA was quickly isolated based on the rat brain positioning map. Part of the tissue was fixed in 4% glutaraldehyde solution and used for transmission electron microscopy, and the other part was immediately stored at −80 °C and used for Western blotting.

### 2.6. Cells and Cell Culture

Rat primary astrocytes were cultured according to previously described methods [27,28]. In brief, 24-h-old suckling mice were soaked in 75% ethanol for 5 min before the head was immersed in Dulbecco’s modified Eagle’s medium (DMEM). The BLA was completely dissected and washed with DMEM before the tissues were cut into blocks (1 mm^2^) and placed in cell culture dishes. Next, 2 mL of trypsin and 20 μL of DNase were added to the cell culture dishes, and digestion was carried out in a 37 °C incubator for 30 min. Digestion was terminated with DMEM culture medium containing 10% FBS, and the cells were centrifuged at 235× *g* and room temperature for 5 min. The pellet was retained. Astrocyte culture medium (no. 1801; ScienCell, Carlsbad, CA, USA) was used to resuspend cells. Rat astrocytes were allowed to grow at 37 °C in a 5% CO_2_ incubator. The culture medium was changed twice every week.

In the following experiments, the cultured rat astrocytes were randomly assigned to four groups and cultured under different conditions: In the control group, the cells were maintained in normal DMEM; in the NE group, cells were cultured in culture medium containing 10 μM of NE for 1 h; in the NE + SB203580 group, cells were cultured in culture medium containing 3 µM of SB203580 first, before 10 μM of NE was added and the cells were cultured for 1 h; finally, in the SB203580 group, cells were cultured in culture medium containing 3 µM of SB203580 for 2 h.

### 2.7. Hematoxylin and Eosin (HE) Staining

Paraffin sections were dried at 60 °C for 40 min. After dehydration using an ethanol gradient, HE staining was performed according to the manufacturer’s protocols (G1120; Solarbio, Beijing, China), followed by observation under a light microscope (Olympus IX71; Olympus Corp., Tokyo, Japan).

### 2.8. Transmission Electron Microscopy (TEM)

The BLA tissue was fixed with 2.5% glutaraldehyde for 48 h. After overnight fixation, samples were post-fixed in 1% Osmium tetroxide, dehydrated through graded concentrations of ethanol, infiltrated, and embedded in Epon 812 at 60 °C for 48 h. Ultra-thin sections were cut with a diamond knife, mounted on formvar-coated slot grids, and then stained with 2% uranyl acetate and lead citrate for 15 min. Images were captured with an H-7500 model Hitachi TEM (Hitachi, Tokyo, Japan).

### 2.9. Immunohistochemistry Assay 

The cleared sections were pre-processed using a microwave for antigen retrieval before blocking with goat serum at 37 °C for 40 min. The sections were incubated with anti-AQP4 antibody (1:200, ab259318, Abcam, Cambridge, UK) and anti-glial fibrillary acidic protein (GFAP) antibody (1:100, ARG10122; Arigobio, Shanghai, China) at 4 °C overnight. After that, the sections were incubated with the fluorescent secondary antibodies Dylight-488 (1:200, 46602; Thermo Fisher Scientific, Waltham, MA, USA) and Dylight-594 (1:200, 46608; Thermo Fisher Scientific, Waltham, MA, USA) at 37 °C for 1 h. Finally, DAPI-containing anti-fluorescence quenching medium (0100-20; SouthernBiotech, Birmingham, AL, USA) was used for mounting. A laser confocal microscope (TCS SP8; Leica, Wetzlar, Germany) was used for single-layer scanning of fluorescence images. ImageJ (version 1.8.0; U.S. National Institutes of Health, Bethesda, MD, USA, https://imagej.net/ij/index.html, accessed on 12 June 2023) was used to quantitate fluorescence intensity. The primary antibodies were replaced with 0.01 mmol/L phosphate buffer saline (PBS) in the negative controls.

### 2.10. Immunocytochemistry Assay

Astrocytes cultured on 24-well coverslips were removed from the culture medium. The coverslips were cleaned with 1× PBS twice, and 4% paraformaldehyde was applied for fixation at room temperature for 15 min. Cells were permeabilized in 0.5% Triton-X-100 for 15 min and blocked with normal goat serum for 30 min at room temperature. Primary antibodies against AQP4 (1:200, ab259318; Abcam, Cambridge, UK) and GFAP (1:100, ARG10122; Arigobio, Shanghai, China) were added to the coverslips and left at 4 °C overnight. We performed a negative control to ensure the specific binding of antibodies to target proteins. Then, the coverslips were incubated with the fluorescent secondary antibodies Dylight-488 (1:200; 46602, Thermo Fisher Scientific, Waltham, MA, USA) and Dylight-594 (1:200, 46608; Thermo Fisher Scientific, Waltham, MA, USA) at 37 °C for 1 h. Finally, the coverslips were washed with PBS three times before DAPI-containing anti-fluorescence quenching medium (0100-20, SouthernBiotech, Birmingham, AL, USA) was used for mounting. A laser confocal microscope (TCS SP8; Leica, Wetzlar, Germany) was used for single-layer scanning of fluorescence images. ImageJ (version 1.8.0; U.S. National Institutes of Health, Bethesda, MD, USA, https://imagej.net/ij/index.html, accessed on 12 June 2023) was used for quantitation of the fluorescence intensity. The primary antibodies were replaced with 0.01 mmol/L PBS in the negative controls.

### 2.11. Western Blot Analysis 

The protein levels of GFAP, AQP4, p-p38 MAPK, p38 MAPK, β-actin, and β-tubulin were examined with Western blotting. In brief, the BLA tissue and astrocytes were lysed with RIPA buffer (Solarbio, Beijing, China), supplemented with protease inhibitors (P8340, Sigma-Aldrich, St. Louis, MO, USA) and phosphatase inhibitors (P0044, Sigma-Aldrich, St. Louis, MO, USA), to obtain the total protein. Lysates were centrifuged at 9168× *g* and 4 °C for 15 min, and the supernatant was collected. The concentrations of protein in the supernatant were measured using a bicinchoninic acid (BCA) kit (PC0020, Solarbio, Beijing, China). An equal amount of protein samples was subjected to 10% SDS-PAGE (SW155-02, Seven, Beijing, China) and transferred to PVDF membranes. The membranes were blocked with 5% bovine serum albumin (4240GR, Biofroxx, Einhausen, Germany) in TBST at 37 °C for 1 h, then incubated with primary antibodies against GFAP (1:500; ARG10122, Arigobio, Shanghai, China), AQP4 (1:1000; ab259318, Abcam, Cambridge, UK), p-p38 MAPK (1:1000; 4511, Cell Signaling Technology, Boston, MA, USA), p38 MAPK (1:1000; ab170099, Abcam, Cambridge, UK), β-actin (1:10,000; AC006, ABclonal, Wuhan, China), and β-tubulin (1:1000; 2146, Cell Signaling Technology, Boston, MA, USA), respectively, at 4 °C overnight. They were then incubated with fluorophore-conjugated secondary antibody (1:10,000; C50317-02, Rockland, Philadelphia, PA, USA). The emitted light was detected and analyzed using an Odyssey gel imaging system (LI-COR, Rahway, NJ, USA), and the relative target band intensity was normalized to β-actin using ImageJ (version 1.8.0; U.S. National Institutes of Health, Bethesda, MD, USA, https://imagej.net/ij/index.html, accessed on 12 June 2023).

### 2.12. Astrocyte Morphometry 

The tissue sections were observed and images were captured using a TCS SP8 laser scanning confocal microscope (Leica, Wetzlar, Germany). Images were processed using the ImageJ Fiji plugin (version 1.8.0; U.S. National Institutes of Health, Bethesda, MD, USA, https://imagej.net/ij/index.html, accessed on 12 June 2023). Except for cell counting, a fixed ROI rectangular block was used for the measurements, and individual cells with intact morphologies were selected for measurement. A two-dimensional image was used for cell counting (enumeration criteria: the nucleus can be clearly seen and localized with GFAP in the same cells). First, the image was opened and the polygonal tool was selected for manual cell counting; then, the image was saved and the cell count was recorded. Rat primary astrocyte cytoskeletons were analyzed according to previously described methods [29]. In brief, fluorescence images were converted to binary images before converting them to cytoskeleton images for measurement. The astrocyte volume and area were analyzed according to previously described methods [30]. Three-dimensional Sholl analysis was similarly performed according to previously described methods [31]; briefly, the Sholl analysis plugin of Fiji was used to analyze the number of astrocyte branches. During software analysis, the length from the filament center to the filament end was taken to be the radius of the total measurement. We constructed concentric spheres with increasing radii (from 6 to 33 μm with 3 μm steps) from the center of the astrocyte soma and extracted different parameters by intersecting spheres with the branch map for each cell. The data obtained were statistically analyzed using GraphPad Prism (version 9.5.0; GraphPad Software, San Diego, CA, USA, https://www.graphpad.com, accessed on 12 June 2023).

### 2.13. Statistical Analysis 

Using the Kolmogorov–Smirnov test method, the data were found to follow a normal distribution among all groups (*p* > 0.1). The data are given as means ± SEM. A two-tailed Student’s *t*-test was used for comparison of means between groups, while analysis of variance (ANOVA) was used for comparisons of ≥3 groups, followed by a Bonferroni post hoc test. The differences were considered significant at *p* < 0.05. GraphPad Prism was used for statistical analysis and graphing.

## 3. Results

### 3.1. Stress Induces BLA Astrocyte Cytotoxic Edema in Rats

**Figure 2:**
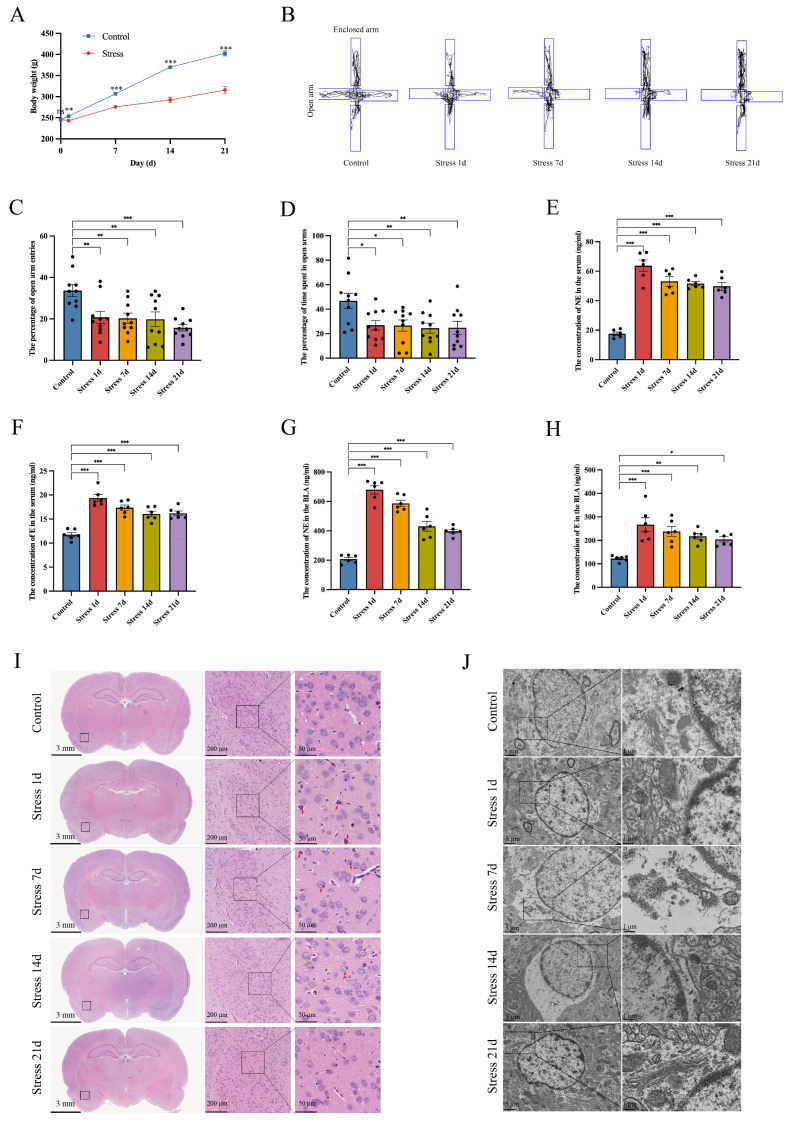
Stress induces anxiety-like behavior and BLA astrocyte cytotoxic edema in rats. (**A**) Weight changes in rats under different stress durations (*n* = 10 per group); (**B**) EPM movement trajectory of rats under different stress durations; (**C**) percentage of open-arm entries on the EPM; (**D**) percentage of time spent in open arms of the EPM; (**E**) serum NE concentrations of rats under different stress durations (*n* = 6 per group); (**F**) serum E concentrations of rats under different stress durations (*n* = 6 per group); (**G**) BLA NE concentrations of rats under different stress durations (*n* = 6 per group); (**H**) BLA E concentrations of rats under different stress durations; (**I**) representative HE staining images for each group (*n* = 5 per group). The yellow arrows indicate edema of cells and red arrows indicate neuronal death. Scale bar from left to right: 3 mm; 200 µm; 50 µm; and (**J**) representative BLA transmission electron microscopy images of rats under different stress durations (*n* = 5 per group). Scale bar from left to right: 5 µm; 1 µm. Data presented as mean ± SEM. ns, no significance. * *p* < 0.05, ** *p* < 0.01, and *** *p* < 0.001 between the indicated groups. EPM: elevated plus maze; NE: noradrenaline; E: adrenaline; HE: hematoxylin–eosin.

During the stress period, the rate of weight gain in rats in the stress group was slower than that of rats in the control group (Figure 2A). The elevated plus maze test (EPM) is a standard and effective method for testing anxiety behaviors in rodents [32,33]. During RS, we found that the percentage of entries in the open arm and the percentage of time spent in the open arm were significantly decreased in the stress group compared to the control group (Figure 2B–D). These results indicated that stress can cause anxiety in rats. In addition, neuroendocrine responses were abnormal during stress, which can activate the sympathetic nervous system and increase stress hormone release [34,35]. We used ELISA to measure serum NE and E levels in rats. The results showed that, compared to the control group, both serum NE and E levels were significantly increased in the stress 1-day group, which decreased with increasing restraint duration, but were still higher than those in the control group (Figure 2E,F). At the same time, we measured the NE and E levels in rat BLA astrocytes. It was found that the levels of BLA NE and E in the stress groups were significantly higher than those in the control group (Figure 2G,H). Overall, the observed behavioral abnormalities and elevated hormone levels indicated successful construction of the rat stress model. In order to observe BLA damage in rats under stress in general, we employed histological staining and transmission electron microscopy. The HE staining results indicated neuronal necrosis and cellular edema in the BLA of rats in the stress groups (Figure 2I). In addition, transmission electron microscopy imaging of the BLA of rats in the stress groups showed astrocyte edema, mitochondrial and Golgi body edema, blurry membrane borders, loose cytoplasmic structure, and even complete disappearance of organelles (Figure 2J). This is consistent with the results of previous studies [17,36], including neuronal mitochondrial membrane rupture, edema, and fusion/fission defect (fusion into huge mitochondria or fission into small mitochondria); edematous vesicles in Golgi bodies; and an increased number of lysosomes seen on transmission electron microscopy images (Figure S1).

### 3.2. Stress Up-Regulates BLA AQP4 Expression in Rats

**Figure 3:**
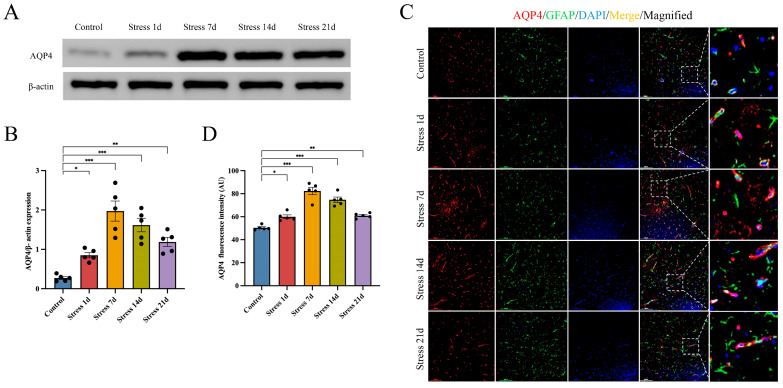
Effects of rat stress on AQP4 expression in rat BLA: (**A**,**B**) Western blot and quantitative analysis of BLA AQP4 protein levels of rats under different stress durations (*n* = 5 per group); (**C**) representative immunofluorescent images of AQP4 (red) and GFAP (green) in the BLA of rats with different durations of stress. Scale bar: 100 µm. (**D**) The mean fluorescence intensity of AQP4 per section was quantified (*n* = 5 per group). Data presented as mean ± SEM. * *p* < 0.05, ** *p* < 0.01, and *** *p* < 0.001 versus control group. AQP4: aquaporin-4; GFAP: glial fibrillary acidic protein.

AQP4 is the most highly expressed aquaporin in brain tissues, which is mainly expressed in astrocytes; in particular, AQP4 is the most critical aquaporin in astrocytes [37]. In order to study the expression level of AQP4 in BLA after exposure of rats to stress, Western blotting was performed. Compared with the control group, the BLA AQP4 expression was significantly higher in the RS 1-day group and was the highest in the 7-day group. Although AQP4 expression started to decrease in RS rats with increasing restraint duration, its expression level remained higher than that in the control group (Figure 3A,B). Immunofluorescence staining also showed that AQP4 presented an increasing trend when assessed at 7, 14, and 21 days of stress (Figure 3C,D). Interestingly, the changes in the AQP4 expression level were generally consistent with the cellular edema revealed through transmission electron microscopy. These results suggest that the increase in the AQP4 expression level could participate in astrocyte edema in rat BLA.

### 3.3. NE Down-Regulation Inhibits Stress-Induced BLA Astrocyte Cytotoxic Edema in Rats

**Figure 4:**
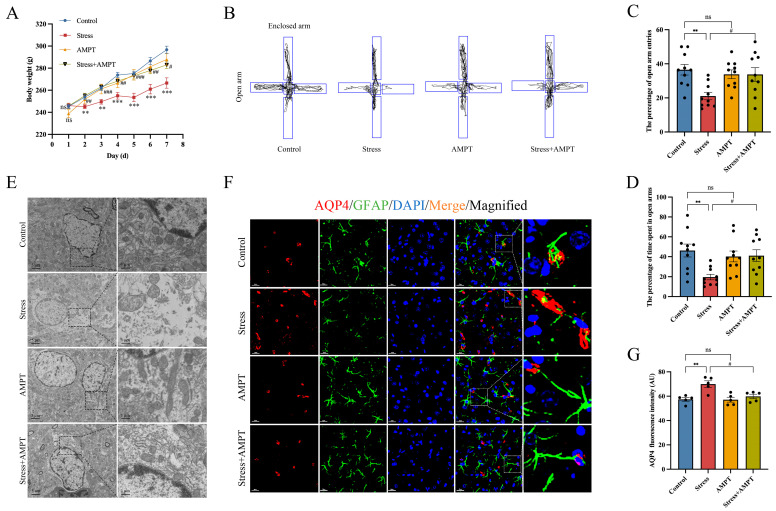
NE inhibition alleviates stress-induced anxiety-like behavior and BLA astrocyte cytotoxic edema in rats. (**A**) Effects of the NE inhibitor AMPT (100 mg/kg) on rat weight (*n* = 10 per group); (**B**–**D**) effects of the NE inhibitor AMPT (100 mg/kg) on stress-induced anxiety-like behavior in rats (*n* = 10 per group); (**E**) representative transmission electron microscopy images of rat BLA (*n* = 5 per group). Scale bar from left to right: 5 µm; 1 µm. (**F**) Representative immunofluorescence images of AQP4 (red) and GFAP (green) in the BLA of rats. Scale bar: 20 µm. (**G**) The mean fluorescence intensity of AQP4 per section was quantified (*n* = 5 per group). Data presented as mean ± SEM. ns, no significance. ** *p* < 0.01 and *** *p* < 0.001 versus control group; ^#^ *p* < 0.05, ^##^ *p* < 0.01, and ^###^ *p* < 0.001 versus stress group. AMPT: alpha-methyl-p-tyrosine.

**Figure 5:**
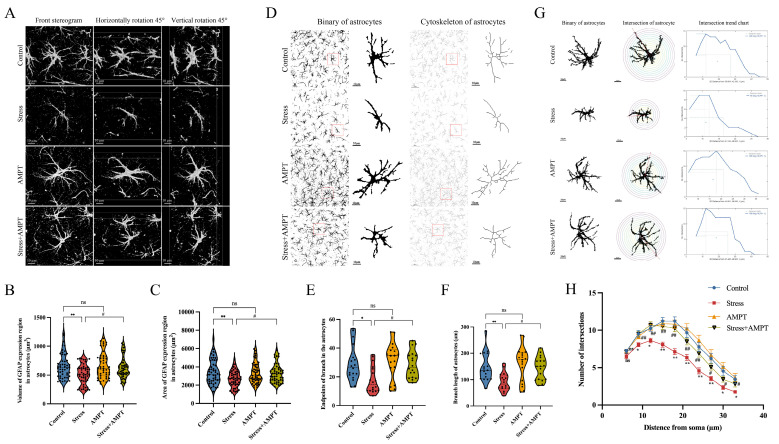
NE inhibition alleviates stress-induced structural remodeling in astrocytes. (**A**–**C**) Comparison of BLA astrocyte GFAP expression region volume and area in rats from different groups (*n* = 50 per group). Scale bar: 10 µm. (**D**) Representative binary images of BLA astrocytes and cytoskeleton images. Scale bar from left to right: 50 µm; 10 µm. (**E**) Endpoints of branches in the astrocytes (*n* = 18 per group). (**F**) Branch length of astrocytes (*n* = 18 per group). Sholl analysis and representative astrocyte vectors are depicted in (**G**). Scale bar: 10 µm. (**H**) Sholl analysis of BLA astrocytes in rats exposed to stress, illustrating the number of intersections found in astrocytes (*n* = 50 per group). Data presented as mean ± SEM. ns, no significance. * *p* < 0.05 and ** *p* < 0.01 versus control group; ^#^ *p* < 0.05 and ^##^ *p* < 0.01 versus stress group.

We hypothesized that NE may play an important role in stress-induced BLA astrocyte cytotoxic edema in rats. Therefore, the NE inhibitor AMPT was used to inhibit NE synthesis before stress exposure. AMPT was tested for in vivo therapeutic efficacy, and our study results indicated that AMPT pre-treatment could reverse the inhibitory effects of stress on weight gain in rats (Figure 4A). As a reflection of anxiety-like behavior in stressed rats, the percentage of entries in the open arm and the percentage of time spent in the open arm were significantly increased in stressed rats (Figure 4B–D). In addition, transmission electron microscopy showed that AMPT significantly alleviated edema in BLA astrocytes (Figure 4E). Next, immunofluorescence staining was performed to assess the fluorescence intensity of AQP4 in the BLA of rats. The stress group showed moderate immunoreactivity to AQP4, while the Stress + AMPT group presented a significant decrease in AQP4 fluorescence intensity (Figure 4F,G). GFAP is a cytoskeleton protein in astrocytes which participates in the cytoskeleton composition and maintenance of cytoskeleton tensile strength [38]. We extracted individual astrocyte images for precise analysis. Likewise, we found that AMPT significantly inhibited the reductions in GFAP area and the volume increase in rat astrocytes induced by stress (Figure 5A–C), suggesting that astrocyte edema was alleviated. Further analysis showed that the number of cytoskeleton branches and their lengths were significantly decreased in BLA astrocytes from stressed rats compared to rats in the control group, providing solid data to demonstrate that stress induced astrocyte edema. However, the stress-induced reductions in the number of cytoskeleton branches and branch length were rescued with AMPT (Figure 5D–F). In addition, our Sholl analysis results of the astrocytes showed that the number of nodes within the micro-measurement radius was decreased in the stress group compared to that in the control group. However, AMPT effectively inhibited this decrease in the number of astrocyte branches in the stressed rats (Figure 5G,H). These results suggest that AMPT pre-treatment can inhibit stress-induced cytotoxic edema and structural remodeling in astrocytes.

### 3.4. NE Treatment Triggers Cytotoxic Edema and Enhances the Expression of AQP4 in Cultured Astrocytes

**Figure 6:**
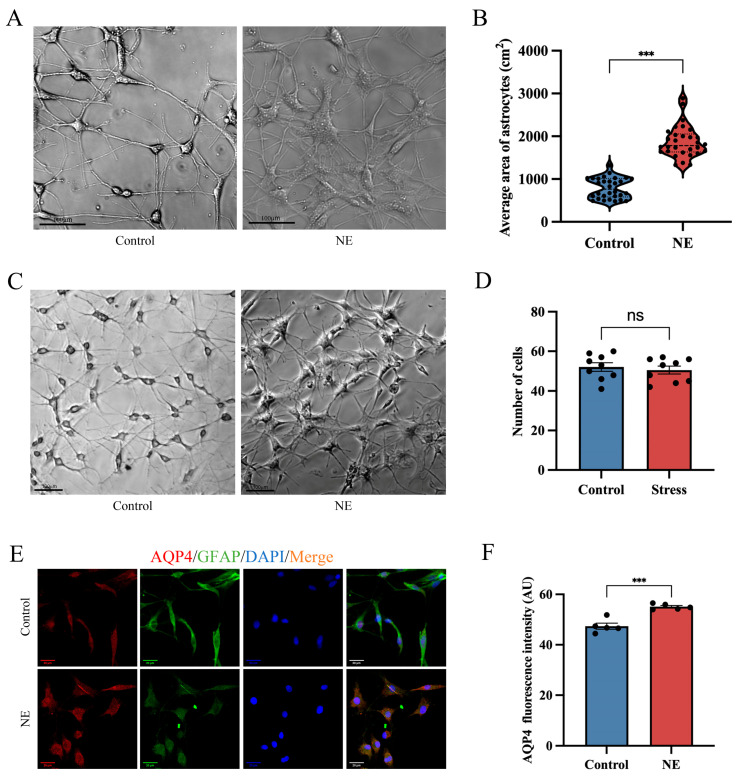
NE treatment triggers cytotoxic edema and enhances the expression of AQP4 in cultured astrocytes. Rat astrocytes were incubated with control DMEM or 10 μM of NE for 1 h. (**A**) Representative original images of rat astrocytes. Scale bar: 100 µm. (**B**) Change in area of cultured astrocytes (*n* = 30 per group). (**C**) Representative original images of rat astrocytes. Scale bar: 100 µm. (**D**) Change in number of cultured astrocytes (*n* = 9 per group). (**E**) Representative immunofluorescence images of AQP4 (red) and GFAP (green) in the cultured astrocytes. Scale bar: 20 µm. (**F**) The mean fluorescence intensity of AQP4 per section was quantified (*n* = 5 per group). Data presented as mean ± SEM. ns, no significance. *** *p* < 0.001 versus control group. DMEM: Dulbecco’s modified Eagle’s medium.

To confirm the in vivo data, we cultured primary rat cortical astrocytes in vitro using NE. NE treatment is an effective method for simulating stress in vitro [39,40]. First, microscope photography and GFAP immunofluorescence staining were carried out to identify and isolate primary rat cortical astrocytes. When the isolated cortical astrocytes were cultured for around 14 days, inverted microscopy observation revealed that individual cells were bipolar or multipolar, protrusions were extended, and the cell body sizes were uneven, which meant that the cells had matured at this point (Figure S2A). Immunofluorescence staining indicated that 95% of astrocytes were immunoreactive for GFAP (Figure S2B), which allowed for the determination that they could be used in subsequent studies. Subsequently, we exposed astrocytes to 10 µM of NE for 1 h. Compared with the control group, the area of astrocytes increased (Figure 6A,B), suggesting that edema had occurred. However, there was no change in the astrocyte count (Figure 6C,D). Immunofluorescence staining analysis showed that AQP4 expression was increased in astrocytes stimulated with 10 µM of NE for 1 h compared with the control group (Figure 6E,F). The above results verify that NE stimulation in astrocytes induced edema and increased AQP4 expression in vitro.

### 3.5. p38 MAPK Pathway Inactivation Weakens NE-Mediated Cytotoxic Edema in Astrocytes

**Figure 7:**
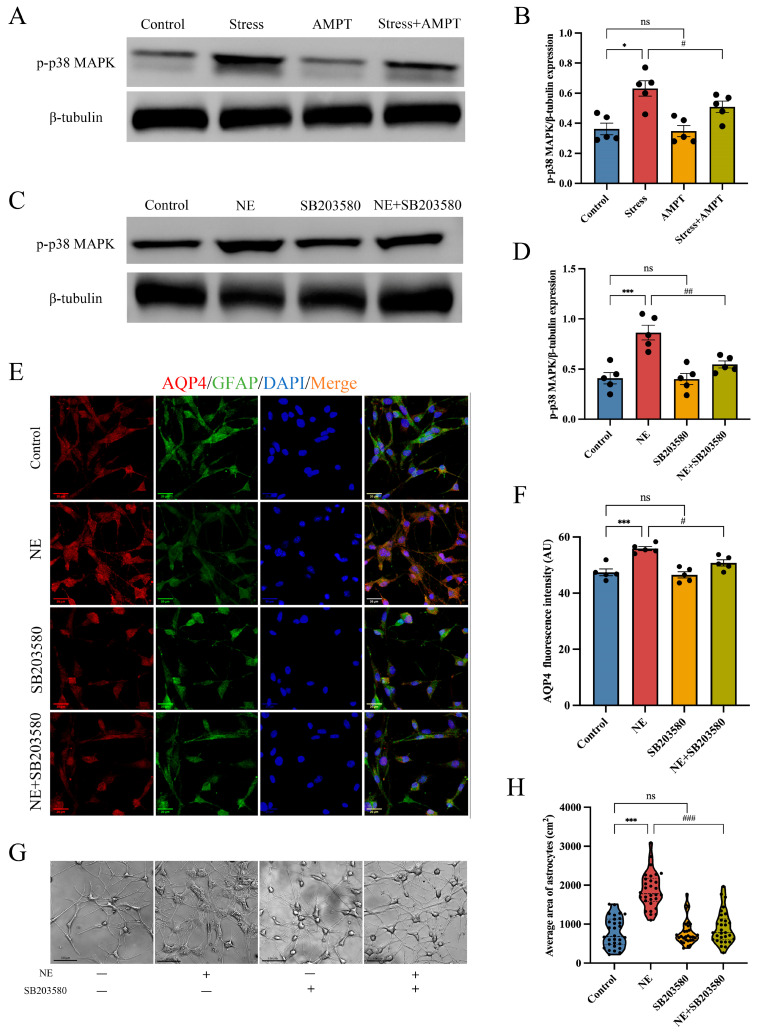
Inhibition of the p38 MAPK pathway reverses NE-mediated astrocyte cytotoxic edema: (**A**) The expression of p38 MAPK pathway activation marker p-p38 MAPK was detected with Western blot in stressed rat BLA. (**B**) Results are expressed as a grayscale ratio of p-p38 MAPK/β-tubulin (*n* = 5 per group). Astrocytes were pre-incubated with 10 μM NE for 1 h, stimulated with 3 µM SB203580 for 1 h, or provided combined treatment. (**C**,**D**) The protein levels of p-p38 MAPK and β-tubulin were detected with Western blot. Quantification of band intensity was performed for each Western blot (*n* = 5 per group). (**E**) Representative immunofluorescence images of AQP4 (red) and GFAP (green) in the cultured astrocytes. Scale bar: 20 µm. (**F**) The mean fluorescence intensity of AQP4 per section was quantified (*n* = 5 per group). (**G**) Representative original images of rat astrocytes. Scale bar: 100 µm. (**H**) Changes in area of cultured astrocytes (*n* = 30 per group). Data presented as mean ± SEM. ns, no significance. * *p* < 0.05 and *** *p* < 0.001 versus control group; ^#^ *p* < 0.05, ^##^ *p* < 0.01, and ^###^ *p* < 0.001 versus stress group and NE group. p38 MAPK: p38 mitogen-activated protein kinase.

The p38 MAPK signaling pathway plays a vital role in regulating the expression of aquaporins [41,42]. Increased p38 phosphorylation is a classical hallmark of p38 MAPK signaling pathway activation. We observed that p38 MAPK phosphorylation was up-regulated in the BLA of stressed rats (Figure 7A,B). Interestingly, when NE was inhibited, p38 MAPK phosphorylation was significantly decreased in the BLA of stressed rats (Figure 7A,B); however, the total protein level of p38 MAPK presented no significant changes (Figure S3A,B). At the cellular level, p38 MAPK phosphorylation was increased after astrocytes were treated with 10 μM of NE for 1 h compared with the control group (Figure 7C,D), which was consistent with the in vivo results. However, there was no significant difference in astrocytes regarding the total protein level of p38 MAPK (Figure S4A,B). To the contrary, as a specific inhibitor of the p38 MAPK pathway, SB203580 was used to treat astrocytes, which significantly inactivated p38 MAPK signal transduction (Figure 7C,D). More importantly, SB203580 significantly alleviated NE-induced astrocyte edema and down-regulated the expression of AQP4 (Figure 7E–H). These results suggest that NE activated the p38 MAPK signaling pathway, mediating cytotoxic edema in stress-induced BLA astrocytes.

## 4. Discussion

Long-term persistent stress or short-term intense stress have negative effects on an individual’s emotions and cognitive function. When the body experiences adverse stimuli, the neuroendocrine response is the basis for metabolic and multi-organ functional changes. Among these changes, the most important neuroendocrine response is activation of the locus coeruleus–sympathetic–adrenomedullary system. Our previous studies have shown that excessive stress could induce neuron degeneration and death, neurotransmitter imbalance, and increased blood–brain barrier permeability, the underlying mechanism of which is related to NE [16,17]. Therefore, the present study focused on the effects and related mechanism of NE on astrocyte edema. Astrocyte foot processes, cerebrovascular endothelial cells, and tight junctions between adjacent endothelial cells surrounding the cells and matrix together constitute the blood–brain barrier [43]. In addition to close contact with brain microvessels, a large number of astrocyte foot processes surround synapses and extend to the surface of the cerebral ventricle to form the glial limiting membrane, which together constitute the structural basis for the regulation of fluid in the brain. The normal morphology and function of astrocytes are crucial for maintaining the brain water balance [44]. Morphological changes in astrocytes are a common pathological characteristic of many neurological diseases, including Alzheimer’s disease [45], Parkinson’s disease [46], and Huntington’s disease [47]. This study examined the relationship between NE and BLA astrocyte injury, and the obtained results suggest that astrocytes are a potential therapeutic target for treating stress-related neuropsychiatric diseases. 

First, in the present study, anxiety-like behavior, increased stress hormone levels, and BLA astrocyte edema were observed in in RS rats, suggesting that restraint stress may induce astrocyte edema. Stress can lead to intracranial neurotransmitter imbalances [48]. Our results showed that the NE level was elevated in the serum and BLA of RS rats. In order to assess whether NE plays an important role in stress-induced BLA astrocyte cytotoxic edema in rats, AMPT was used to inhibit NE synthesis. The experimental results indicated that AMPT not only significantly decreased anxiety-like behavior, but also improved edema and structural remodeling in the BLA astrocytes of stressed rats. Swollen astrocytes do not take in excitatory neurotransmitters, but instead release excitatory neurotransmitters extracellularly [49]. Increases in extracellular excitatory neurotransmitters can trigger neuron hyperactivation, causing excitotoxicity and resulting in a vicious cycle. These results indicate that NE plays an important role in mediating the cytotoxic edema of astrocytes and associated pathological changes. This may explain the underlying mechanism by which overactivation of the sympathetic nervous system induces an imbalance of homeostasis in BLA during stress.

Astrocytes contain many AQPs, and cytotoxic edema in astrocytes is intimately associated with AQPs [10]. AQPs are highly expressed in astrocytes, and their selective pores play a vital role in the maintenance of water and ion homeostasis [50]. AQP4 is mainly expressed in the foot processes of astrocytes, blood vessels, and subarachnoid space, and thus plays a decisive role in the production of cerebral spinal fluid and maintenance of the cerebral spinal fluid balance and ventricular compliance [51]. A previous study [52] showed that AQP4 promoted cytotoxic edema in astrocytes, playing a critical role in the pathogenesis of traumatic brain injury. In our study, AQP4 expression was increased in the cell membrane of BLA astrocytes from stressed rats and peaked at day 7 of restraint stress, which is consistent with the time point at which edema occurred. We also found that the NE inhibitor AMPT down-regulated the expression of AQP4 induced by stress.

In order to observe the adverse effects of stress on astrocytes cultured in vitro, NE was used to simulate astrocyte stress. In vitro, NE exposure induced significant edema and increased AQP4 expression in astrocytes. These data suggest that NE may promote cytotoxic edema in astrocytes under stress conditions. 

Subsequently, we examined the potential mechanisms by which the NE-mediated stress induced cytotoxic edema in astrocytes. p38 MAPK is an important member of the MAPK family which participates in various intracellular signal transductions. p38 MAPK plays an important role in cell growth, differentiation, death, response to external stimuli, and inflammatory responses [21]. A previous study [53] reported that inhibition of p38 MAPK signaling could decrease AQP4 expression, which plays an important role in cerebral edema in traumatic brain injury. Elevated p38 MAPK phosphorylation is a classical hallmark of p38 MAPK signaling pathway activation. In the present study, we found that p38 MAPK phosphorylation was up-regulated in the BLA of stressed rats. In addition, p38 MAPK phosphorylation is also increased in NE-treated primary rat astrocytes. We also found that p38 MAPK phosphorylation was significantly decreased in the BLA of stressed rats when NE was inhibited. These results indicated that the p38 MAPK signaling pathway was activated by NE, both in vivo and in vitro. Subsequently, we found that NE-induced p38 MAPK signaling activation in primary rat astrocytes was blunted when they were treated with SB203580—a specific inhibitor of p38 MAPK signaling—which decreased NE-induced AQP4 elevation. More importantly, SB203580 significantly alleviated NE-induced cytotoxic edema in primary rat astrocytes.

## 5. Conclusions

In summary, our results show that NE content increased, astrocyte cytotoxic edema occurred, and AQP4 expression was up-regulated in BLA obtained from RS rats. In addition, NE treatment triggered cytotoxic edema and enhanced the expression of AQP4 in cultured astrocytes. Pre-treatment with the NE inhibitor AMPT significantly alleviated edema in astrocytes and down-regulated the expression of AQP4 in BLA. When NE was inhibited, we observed that p38 MAPK phosphorylation was significantly decreased in the BLA of stressed rats. More importantly, SB203580 significantly alleviated NE-induced astrocyte edema and decreased NE-induced AQP4 elevation. These results suggest that NE-induced activation of the p38 MAPK pathway could mediate cytotoxic edema in stress-induced BLA astrocytes.

## Data Availability

The original contributions presented in this study are included in the article/supplementary material; further inquiries can be directed to the corresponding authors.

## Supplementary Materials

The following supporting information can be downloaded at: https://www.mdpi.com/article/10.3390/brainsci14020161/s1.

## References

[1] Galluzzi L., Yamazaki T., Kroemer G. (2018). Linking cellular stress responses to systemic homeostasis. Nat. Rev. Mol. Cell Biol..

[2] Timmer-Murillo S.C., Schramm A., deRoon-Cassini T.A. (2022). Life threat during assaultive trauma: Critical posttraumatic stress disorder risk factors for injured patients. J. Trauma. Acute Care Surg..

[3] Zhang J.Y., Liu T.H., He Y., Pan H.Q., Zhang W.H., Yin X.P., Tian X.L., Li B.M., Wang X.D., Holmes A. (2019). Chronic Stress Remodels Synapses in an Amygdala Circuit-Specific Manner. Biol. Psychiatry.

[4] Welcome M.O., Mastorakis N.E. (2020). Stress-induced blood brain barrier disruption: Molecular mechanisms and signaling pathways. Pharmacol. Res..

[5] Turner A.I., Smyth N., Hall S.J., Torres S.J., Hussein M., Jayasinghe S.U., Ball K., Clow A.J. (2020). Psychological stress reactivity and future health and disease outcomes: A systematic review of prospective evidence. Psychoneuroendocrinology.

[6] Liu W.Z., Zhang W.H., Zheng Z.H., Zou J.X., Liu X.X., Huang S.H., You W.J., He Y., Zhang J.Y., Wang X.D. (2020). Identification of a prefrontal cortex-to-amygdala pathway for chronic stress-induced anxiety. Nat. Commun..

[7] Ma H., Li C., Wang J., Zhang X., Li M., Zhang R., Huang Z., Zhang Y. (2021). Amygdala-hippocampal innervation modulates stress-induced depressive-like behaviors through AMPA receptors. Proc. Natl. Acad. Sci. USA.

[8] Zhang X., Kim J., Tonegawa S. (2020). Amygdala Reward Neurons Form and Store Fear Extinction Memory. Neuron.

[9] Kim J., Kang S., Choi T.Y., Chang K.A., Koo J.W. (2022). Metabotropic Glutamate Receptor 5 in Amygdala Target Neurons Regulates Susceptibility to Chronic Social Stress. Biol. Psychiatry.

[10] Zhou Z., Zhan J., Cai Q., Xu F., Chai R., Lam K., Luan Z., Zhou G., Tsang S., Kipp M. (2022). The Water Transport System in Astrocytes-Aquaporins. Cells.

[11] Lafrenaye A.D., Simard J.M. (2019). Bursting at the Seams: Molecular Mechanisms Mediating Astrocyte Swelling. Int. J. Mol. Sci..

[12] Li Y., Li L., Wu J., Zhu Z., Feng X., Qin L., Zhu Y., Sun L., Liu Y., Qiu Z. (2020). Activation of astrocytes in hippocampus decreases fear memory through adenosine A(1) receptors. Elife.

[13] Park M.W., Cha H.W., Kim J., Kim J.H., Yang H., Yoon S., Boonpraman N., Yi S.S., Yoo I.D., Moon J.S. (2021). NOX4 promotes ferroptosis of astrocytes by oxidative stress-induced lipid peroxidation via the impairment of mitochondrial metabolism in Alzheimer’s diseases. Redox Biol..

[14] Fan J., Guo F., Mo R., Chen L.Y., Mo J.W., Lu C.L., Ren J., Zhong Q.L., Kuang X.J., Wen Y.L. (2023). O-GlcNAc transferase in astrocytes modulates depression-related stress susceptibility through glutamatergic synaptic transmission. J. Clin. Investig..

[15] Yi S., Chen K., Zhang L., Shi W., Zhang Y., Niu S., Jia M., Cong B., Li Y. (2019). Endoplasmic Reticulum Stress Is Involved in Stress-Induced Hypothalamic Neuronal Injury in Rats via the PERK-ATF4-CHOP and IRE1-ASK1-JNK Pathways. Front. Cell Neurosci..

[16] Xu G., Li Y., Ma C., Wang C., Sun Z., Shen Y., Liu L., Li S., Zhang X., Cong B. (2019). Restraint Stress Induced Hyperpermeability and Damage of the Blood-Brain Barrier in the Amygdala of Adult Rats. Front. Mol. Neurosci..

[17] Wang S., Shi W., Zhang G., Zhang X., Ma C., Zhao K., Cong B., Li Y. (2019). Endoplasmic Reticulum Stress-Mediated Basolateral Amygdala GABAergic Neuron Injury Is Associated with Stress-Induced Mental Disorders in Rats. Front. Cell Neurosci..

[18] Akter S., Sasaki H., Uddin K.R., Ikeda Y., Miyakawa H., Shibata S. (2019). Anxiolytic effects of γ-oryzanol in chronically- stressed mice are related to monoamine levels in the brain. Life Sci..

[19] Benton K.C., Wheeler D.S., Kurtoglu B., Ansari M.B.Z., Cibich D.P., Gonzalez D.A., Herbst M.R., Khursheed S., Knorr R.C., Lobner D. (2022). Norepinephrine activates β(1)-adrenergic receptors at the inner nuclear membrane in astrocytes. Glia.

[20] Plastira I., Bernhart E., Joshi L., Koyani C.N., Strohmaier H., Reicher H., Malle E., Sattler W. (2020). MAPK signaling determines lysophosphatidic acid (LPA)-induced inflammation in microglia. J. Neuroinflammation.

[21] Muslin A.J. (2008). MAPK signalling in cardiovascular health and disease: Molecular mechanisms and therapeutic targets. Clin. Sci..

[22] Tang K., Zhong B., Luo Q., Liu Q., Chen X., Cao D., Li X., Yang S. (2022). Phillyrin attenuates norepinephrine-induced cardiac hypertrophy and inflammatory response by suppressing p38/ERK1/2 MAPK and AKT/NF-kappaB pathways. Eur. J. Pharmacol..

[23] Qi J., Li R.J., Fu L.Y., Liu K.L., Qiao J.A., Yang Y., Yu X.J., Yu J.Y., Li Y., Tan H. (2022). Exercise Training Attenuates Hypertension via Suppressing ROS/MAPK/NF-κB/AT-1R Pathway in the Hypothalamic Paraventricular Nucleus. Nutrients.

[24] Yan H.C., Cao X., Das M., Zhu X.H., Gao T.M. (2010). Behavioral animal models of depression. Neurosci. Bull..

[25] Li Z., Gao C., Peng J., Liu M., Cong B. (2020). Multi-omics analysis of pathological changes in the amygdala of rats subjected to chronic restraint stress. Behav. Brain Res..

[26] Kraeuter A.K., Guest P.C., Sarnyai Z. (2019). The Elevated Plus Maze Test for Measuring Anxiety-Like Behavior in Rodents. Methods Mol. Biol..

[27] Foo L.C., Allen N.J., Bushong E.A., Ventura P.B., Chung W.S., Zhou L., Cahoy J.D., Daneman R., Zong H., Ellisman M.H. (2011). Development of a method for the purification and culture of rodent astrocytes. Neuron.

[28] Zhang L.Y., Hu Y.Y., Zhao C.C., Qi J., Su A.C., Lou N., Zhang M.Y., Li L., Xian X.H., Gong J.X. (2019). The mechanism of GLT-1 mediating cerebral ischemic injury depends on the activation of p38 MAPK. Brain Res. Bull..

[29] Young K., Morrison H. (2018). Quantifying Microglia Morphology from Photomicrographs of Immunohistochemistry Prepared Tissue Using ImageJ. J. Vis. Exp..

[30] Rai D., Dey S., Ray K. (2018). A method for estimating relative changes in the synaptic density in Drosophila central nervous system. BMC Neurosci..

[31] Popov A., Brazhe A., Denisov P., Sutyagina O., Li L., Lazareva N., Verkhratsky A., Semyanov A. (2021). Astrocyte dystrophy in ageing brain parallels impaired synaptic plasticity. Aging Cell.

[32] Hu C., Luo Y., Wang H., Kuang S., Liang G., Yang Y., Mai S., Yang J. (2017). Re-evaluation of the interrelationships among the behavioral tests in rats exposed to chronic unpredictable mild stress. PLoS ONE.

[33] Yin F., Guo H., Cui J., Shi Y., Su R., Xie Q., Chang J., Wang Y., Lai J. (2019). The basolateral amygdala regulation of complex cognitive behaviours in the five-choice serial reaction time task. Psychopharmacology.

[34] Seo D.O., Zhang E.T., Piantadosi S.C., Marcus D.J., Motard L.E., Kan B.K., Gomez A.M., Nguyen T.K., Xia L., Bruchas M.R. (2021). A locus coeruleus to dentate gyrus noradrenergic circuit modulates aversive contextual processing. Neuron.

[35] Wyrofsky R.R., Reyes B.A.S., Zhang X.Y., Bhatnagar S., Kirby L.G., Van Bockstaele E.J. (2019). Endocannabinoids, stress signaling, and the locus coeruleus-norepinephrine system. Neurobiol. Stress..

[36] Wang S., Liu X., Shi W., Qi Q., Zhang G., Li Y., Cong B., Zuo M. (2021). Mechanism of Chronic Stress-Induced Glutamatergic Neuronal Damage in the Basolateral Amygdaloid Nucleus. Anal. Cell Pathol..

[37] Filippidis A.S., Carozza R.B., Rekate H.L. (2016). Aquaporins in Brain Edema and Neuropathological Conditions. Int. J. Mol. Sci..

[38] Kim K.Y., Shin K.Y., Chang K.A. (2023). GFAP as a Potential Biomarker for Alzheimer’s Disease: A Systematic Review and Meta-Analysis. Cells.

[39] Cao M., Huang W., Chen Y., Li G., Liu N., Wu Y., Wang G., Li Q., Kong D., Xue T. (2021). Chronic restraint stress promotes the mobilization and recruitment of myeloid-derived suppressor cells through β-adrenergic-activated CXCL5-CXCR2-Erk signaling cascades. Int. J. Cancer.

[40] Liu W., Wang X., Gong J., Mei Z., Gao X., Zhao Y., Ma J., Qian L. (2014). The stress-related hormone norepinephrine induced upregulation of Nix, contributing to ECM protein expression. Cell Stress. Chaperones.

[41] Wang Q., Wu Z.L., Yuan X., Dong H.Y., Xu X., Xin H., Wang Y.H., Zhang J.B., Chen L., Li H.L. (2019). Bilobetin induces kidney injury by influencing cGMP-mediated AQP-2 trafficking and podocyte cell cycle arrest. Phytomedicine.

[42] Wang C., Yan M., Jiang H., Wang Q., He S., Chen J., Wang C. (2018). Mechanism of aquaporin 4 (AQP 4) up-regulation in rat cerebral edema under hypobaric hypoxia and the preventative effect of puerarin. Life Sci..

[43] Preininger M.K., Kaufer D. (2022). Blood-Brain Barrier Dysfunction and Astrocyte Senescence as Reciprocal Drivers of Neuropathology in Aging. Int. J. Mol. Sci..

[44] Nutma E., van Gent D., Amor S., Peferoen L.A.N. (2020). Astrocyte and Oligodendrocyte Cross-Talk in the Central Nervous System. Cells.

[45] Paumier A., Boisseau S., Jacquier-Sarlin M., Pernet-Gallay K., Buisson A., Albrieux M. (2022). Astrocyte-neuron interplay is critical for Alzheimer’s disease pathogenesis and is rescued by TRPA1 channel blockade. Brain.

[46] Wei Z.D., Shetty A.K. (2021). Treating Parkinson’s disease by astrocyte reprogramming: Progress and challenges. Sci. Adv..

[47] Diaz-Castro B., Gangwani M.R., Yu X., Coppola G., Khakh B.S. (2019). Astrocyte molecular signatures in Huntington’s disease. Sci. Transl. Med..

[48] Hwang K.A., Hwang H.J., Hwang Y.J., Kim Y.J. (2020). Mustard Leaf Extract Suppresses Psychological Stress in Chronic Restraint Stress-Subjected Mice by Regulation of Stress Hormone, Neurotransmitters, and Apoptosis. Nutrients.

[49] Norenberg M.D., Rao K.V., Jayakumar A.R. (2005). Mechanisms of ammonia-induced astrocyte swelling. Metab. Brain Dis..

[50] Day R.E., Kitchen P., Owen D.S., Bland C., Marshall L., Conner A.C., Bill R.M., Conner M.T. (2014). Human aquaporins: Regulators of transcellular water flow. Biochim. Biophys. Acta.

[51] Hoshi A., Tsunoda A., Tada M., Nishizawa M., Ugawa Y., Kakita A. (2017). Expression of Aquaporin 1 and Aquaporin 4 in the Temporal Neocortex of Patients with Parkinson’s Disease. Brain Pathol..

[52] Michinaga S., Koyama Y. (2021). Pathophysiological Responses and Roles of Astrocytes in Traumatic Brain Injury. Int. J. Mol. Sci..

[53] Liu B.H., Zhou D., Guo Y., Zhang S., Guo Y.M., Guo T.T., Chen X.Y., Gong Y.N., Tang H.L., Xu Z.F. (2021). Bloodletting Puncture at Hand Twelve Jing-Well Points Relieves Brain Edema after Severe Traumatic Brain Injury in Rats via Inhibiting MAPK Signaling Pathway. Chin. J. Integr. Med..

