# Supplementary material for: Norepinephrine-Activated p38 MAPK Pathway Mediates Stress-Induced Cytotoxic Edema of Basolateral Amygdala Astrocytes
Source: Brain Sci. 2024 Feb 4;14(2):161. doi: 10.3390/brainsci14020161 (PMC10887202; doi:10.3390/brainsci14020161)
Supplement: Supplementary file 1 [file brainsci-14-00161-s001.zip › brainsci-2832343-supplementary.pdf]

Supplementary Material

1 Supplementary Figures

1.1 Supplementary Figure S1

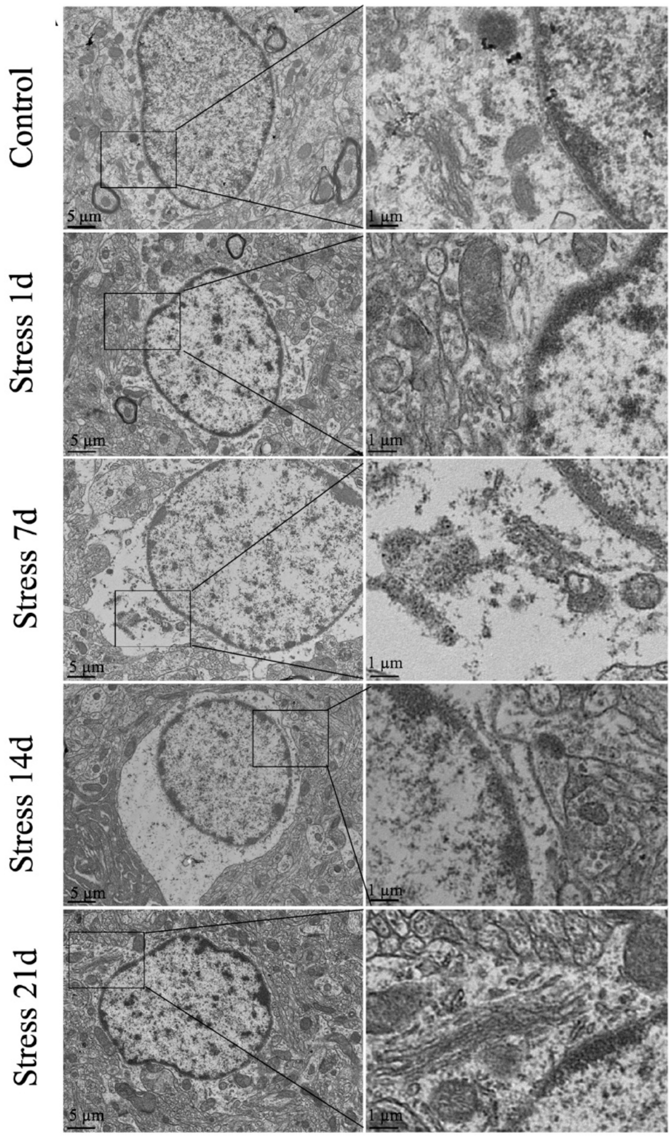

Supplementary Figure S1. Representative BLA neuron transmission electron microscopy images of rats under different stress durations (n = 5 per group). Scale bar from left to right: 5 µm; 1 µm. BLA: basolateral amygdala.

1.2 Supplementary Figure S2

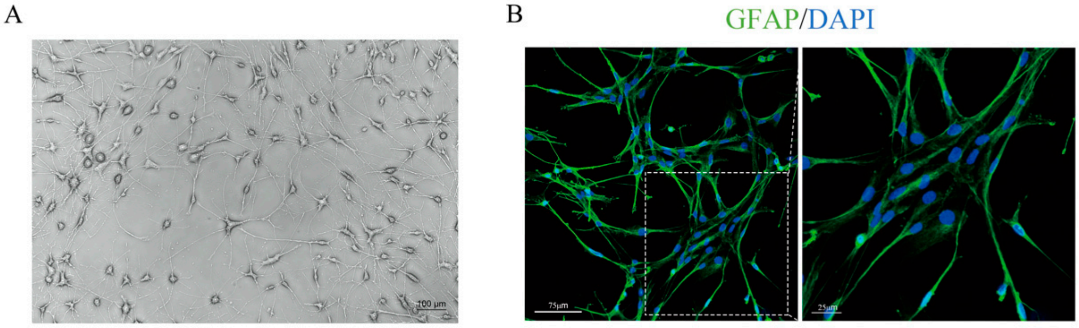

Supplementary Figure S2. Rat amygdala astrocyte culture and identification: (A) Morphological characteristics of rat amygdala astrocytes. Scale bar: 100 μm; and (B) Representative immunofluorescent images of GFAP (green) and DAPI (blue) in the cultured astrocytes. Scale bar from left to right: 75 μm; 25 μm. GFAP: glial fibrillary acidic protein.

1.3 Supplementary Figure S3

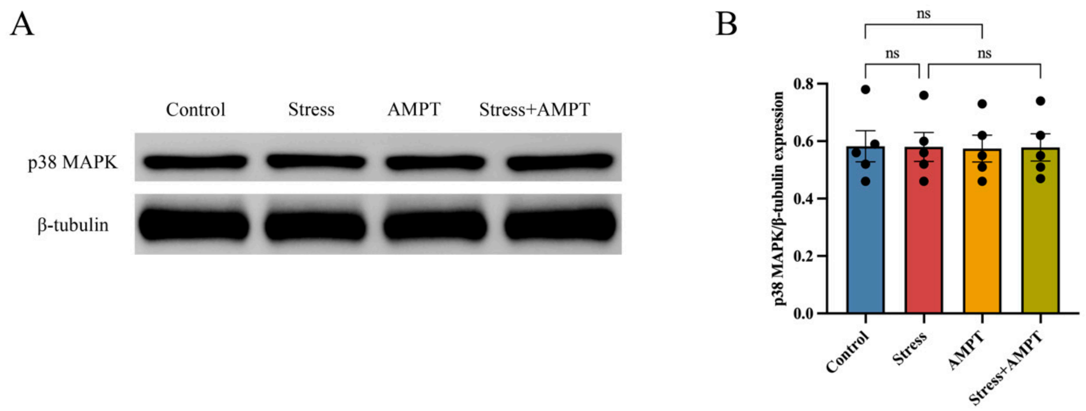

Supplementary Figure S3. Expression of p38 MAPK protein levels in stressed rat BLA: (A) The expression of p38 MAPK detected by Western blot; and (B) Results expressed as grayscale ratio of p38 MAPK to β-tubulin (n = 5 per group). Data presented as mean ± SEM. ns, no significance. p38 MAPK: p38 mitogen-activated protein kinase; AMPT: alpha-methyl-p-tyrosine; BLA: basolateral amygdala.

1.4 Supplementary Figure S4

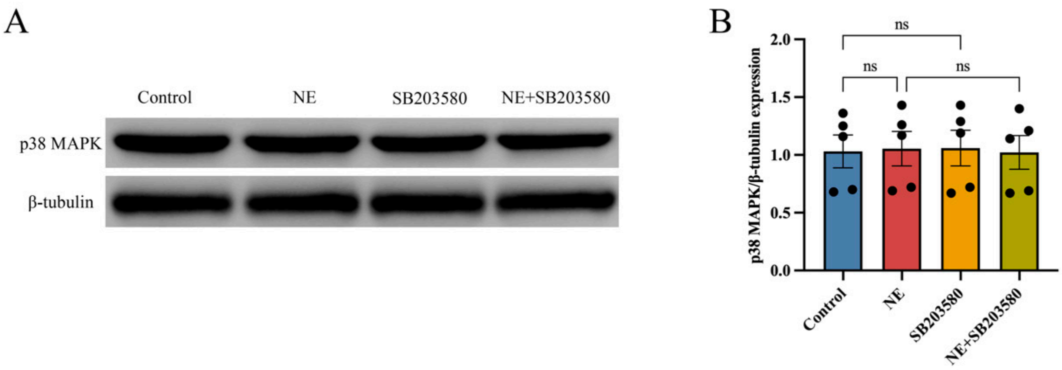

Supplementary Figure S4. Expression of p38 MAPK protein levels in cultured astrocytes: (A) The expression of p38 MAPK was detected by Western blot; and (B) Results expressed as grayscale ratio of p38 MAPK to β-tubulin (n = 5 per group). Data presented as mean ± SEM. ns, no significance. p38 MAPK: p38 mitogen-activated protein kinase; NE: norepinephrine.
